# Marine radar reveals novel group foraging patterns in pelagic seabirds

**DOI:** 10.1098/rstb.2025.0203

**Published:** 2026-10-15

**Authors:** Yann Tremblay

**Affiliations:** MARBEC: IRD - IFREMER - CNRS - University of Montpellier, Sète, Hérault, France

**Keywords:** multi-specific aggregation, flock feeding, social behaviour, collective pattern, aeroecology, chessboard, rake, cluster

## Abstract

Many seabirds are known to frequently feed within multi-specific feeding flocks. These aggregations bring facilitation to prey access as well as potential exacerbated competition. Once a feeding opportunity is found, birds rapidly aggregate to it through processes known as local enhancement. At a larger scale, seabirds may distribute themselves forming patterns such as rakes or clusters, which are thought to increase the probability to cue on other foragers. The importance of such patterns in the foraging ecology of seabirds is yet to be evaluated empirically. Using a large dataset of radar images from the eastern tropical Atlantic, spanning between November 2015 and November 2018, I visually looked for the presence of collective patterns in seabirds' distribution at sea. I showed that clusters, rakes and the newly discovered chessboard patterns were found in 75.3, 68.6 and 51.0% of the recorded days, respectively. Furthermore, these patterns were observed virtually everywhere in the eastern tropical Atlantic and preceded the observations of local enhancement. These results indicate that collective patterns represent a widespread strategy, probably involving many different species associated with subsurface predators. The significance of this ubiquity is discussed in light of the strategic thinking possibly required to maintain these patterns, as well as their evolutionary and conservation implications.

This article is part of the theme issue ‘Multi-predator feeding aggregations in pelagic waters’.

## 1. Introduction

Group formation is a fundamental trait in seabirds. Most seabirds nest in dense colonies and when foraging, they often join feeding aggregations which are typically either composed of conspecific individuals or several distinct species [1–6]. Relying on ‘others’ can considerably enhance prey detection through processes such as local enhancement [7–9] or capture facilitation [10], but this can be detrimental when ‘others’ might be missing owing to biodiversity erosion, putting populations particularly at risk of decline, as suggested by modelling studies [11,12]. Foraging seabirds therefore rely on a mix of personal information (vision, olfaction and memory) and public information (inferences from other predators signalling the presence of prey), in order to make foraging decisions [8,13–16].

The costs (competition for food) and benefits (higher efficiency) of forming groups may depend on a wide variety of factors, such as environmental conditions affecting the availability of prey or the composition of the foraging guild present in the foraging range [17,18]. Indeed, the efficiency of inferring the presence of prey through information gathered from other predators might depend on the availability of such ‘information sources’, the so-called ‘foraging network’ [15,19–21]. The way in which foraging networks are organized is mostly unknown. In order of increasing behavioural complexity, they can arise fortuitously from the common presence of predators in an area, be actively maintained after arising fortuitously or be strategically built, maintained and organized.

Studying foraging networks is particularly challenging, especially in seabirds that forage over vast distances. In recent years, the use of marine radars allowed this challenge to be partially addressed [15,21–23]. Seabirds were observed to form clusters (i.e. medium-scale group associations, approximately 9 km in maximum length, of smaller-scale seabird groups flying together) [21] and to engage in ‘rakes’ (i.e. coordinated alignments of flying seabirds over several kilometres) [15]. These two spatial patterns involve inter-individual distance monitoring and a degree of behavioural coordination, which strongly suggests that foraging networks are a result of strategic foraging decisions. However, until now, the prevalence of such networks and patterns has been largely unexamined, precluding strong conclusions regarding their importance and evolutionary significance.

To better understand the overall significance of foraging networks, I data-mined 97 920 radar images (over the whole eastern tropical Atlantic Ocean) to search for incidences of spatial patterns that suggests strategic collective organization among foraging seabirds. The aim of this study was to document empirically both the prevalence and spatial distribution of these patterns to gain insights into their importance in finding prey.

## 2. Methods

### (a) Data collection

Data were recorded from two French tuna purse-seiners (namely Gueotec and Gueriden) fishing in the tropical Atlantic (Gulf of Guinea) between 21 June 2016 and 22 March 2018, and between 16 November 2015 and 26 November 2018, for the Gueotec and Gueriden respectively, spanning all seasons.

In addition to navigation radar, most industrial tuna purse-seiners operating in tropical oceans are equipped with seabird-dedicated radar. Fishermen scrutinize the seabird-radar's screen in search of seabird–tuna feeding flocks, the presence of which is interpreted from patterns of seabirds' motion in an 8 nautical mile radius circle around the ship (approximately 15 km radius).

To detect seabirds under optimal conditions at all times, fishermen continuously adjust radar settings (e.g. gain and wave filters), thereby maximizing seabird visibility.

Both ships were equipped with FAR-2137 marine surveillance radars (S-band, 30 kW, FURUNO).

Radar screenshot images were recorded as *.png files (i.e. using lossless compression) every 15 seconds onto a laptop computer using an external video card (DVI2USB frame-grabber, Epiphan System Inc., Ottawa, Canada). The radar box was connected to the computer with DVI-D and USB cables (i.e. records were in numeric format).

A Bash script in Linux was coded to automate the recording of images without the need for intervention by the fishermen. This script auto-launched itself at computer startup and automatically detected the presence of a video stream from the video card, in which case the recording occurred at fixed intervals. This script guaranteed the continuity of data recording over several months, as it could handle power outages and the radar being turned on and off. Data were downloaded to an external hard drive every few months when the ship returned to the port of Abidjan (Ivory Coast).

Echo intensities were displayed on screen following a colourmap (chosen by the fishermen as yellow-brownish and red-greenish in the Gueotec and Gueriden, respectively). Colour allows visualization of the strength of an echo. Past echoes or ‘trails’ were blue in both ships (see figure 1).

**Figure 1.**
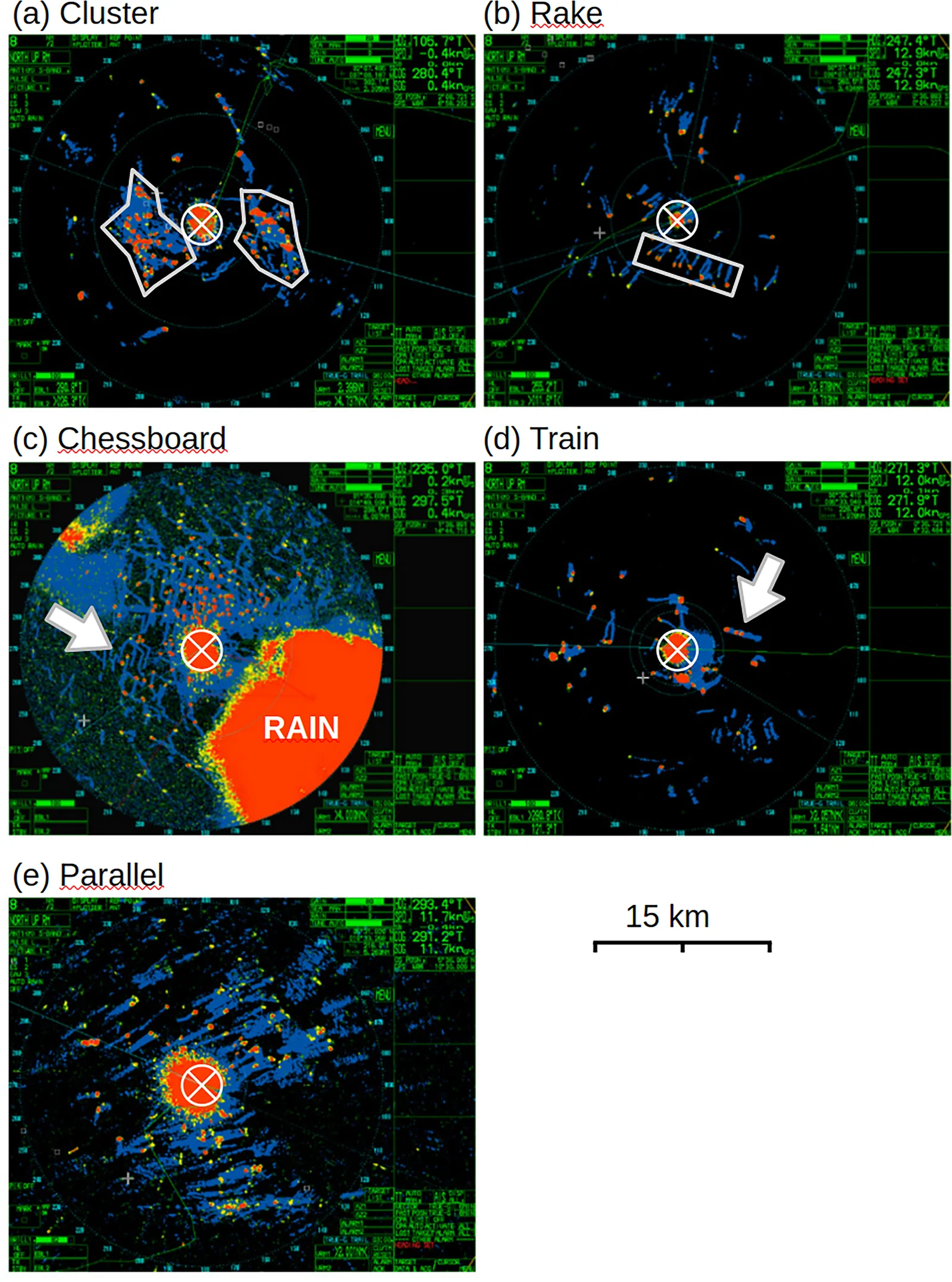
Selected radar images showing examples of seabirds' collective patterns measured in this study. The scale is the same for all panels. A white circled-cross was added to show the location of the boat in the radar image. The white contours in panels (a) and (b) and the white arrows in panels (c) and (d) are used to pinpoint the pattern in the middle of the other echoes. Red/yellow dots indicate echoes. The blue lines show past echoes, i.e. trails, and are used to visualize the direction of movement of the echoes.

### (b) Data processing

The analysis was designed to use a daily resolution.

For each ship, the images were regrouped into daily folders of approximately 5760 files. A mosaic of a regular subset of 240 images was produced for each day with a resolution of 400 × 300 pixels per tile (allowing sufficient detail for detecting echoes and spatial patterns). Mosaics were produced using a custom-built Bash script in Linux that used the ‘montage’ function from the ImageMagick suite of tools (https://imagemagick.org/). Each mosaic thus represented a daily snapshot of radar data with a tile approximately every 6 minutes (figure 2). This interval was chosen because it corresponded to the trail duration chosen by the fishermen on the radar screen. ‘Trail duration’ is a parameter set by the fisherman, allowing for past echoes to be displayed in different colours on the radar screen. This allows for the visualization of tracks over a given period of time. Thus, each tile of the mosaic displayed tracks that occurred in the time elapsed since the previous tile, in blue colour. The subset of images used for the mosaics totalled 116 400 individual images. Examples of mosaics are given in the supplementary material.

**Figure 2.**
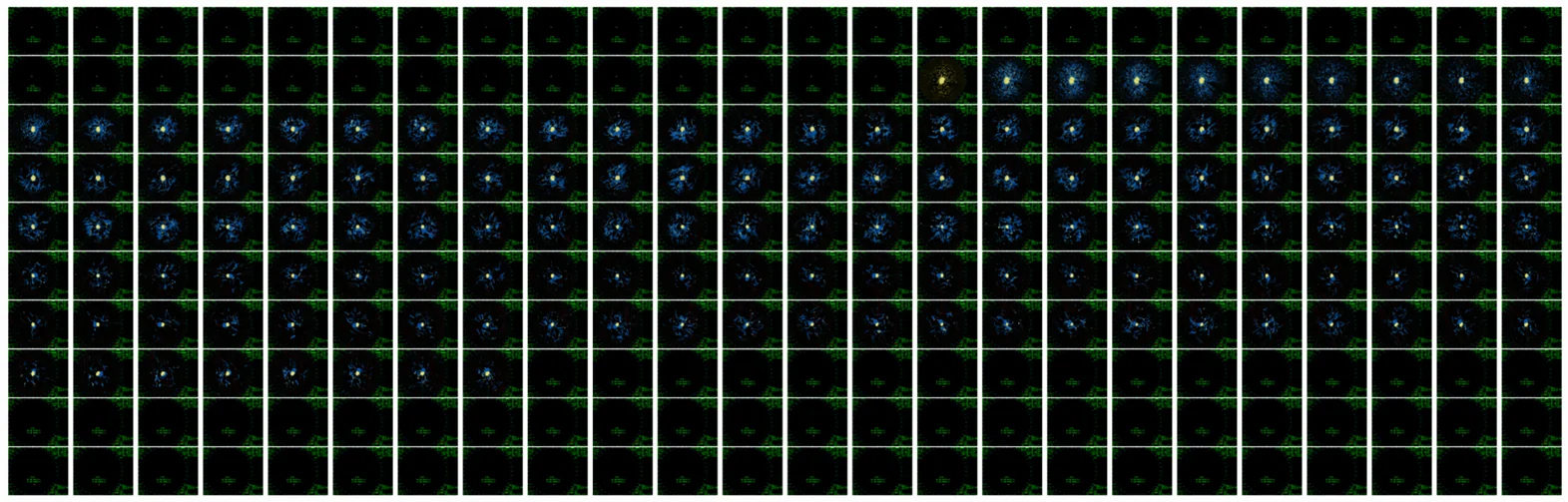
Example of 24H mosaic created to data-mine a huge collection of radar images. Tiles are approximately 6 minutes apart and go from 00:00 in the beginning (upper-left) to 00:00 in the end (lower-right) of the day. Note the daily activity routine of tuna purse-seiners, who turn their radar into standby mode at night.

As the data were automatically recorded, they were screened and removed for days when proper observation was not possible. These included days when ships were in port, days when the radar was always in standby mode, and days with only partial recording or with images of poor quality (owing to heavy rainfall for a long period of time, for example). Data recorded on ‘ideal days’ were retained for subsequent analysis.

The location of each radar image used for the mosaic was read from the radar GPS-location field in the image using a custom-built optical character recognition (OCR) software (Y. Tremblay 2013, unpublished data, based on codes by A. Saroch—MATLAB central file exchange) coded in MATLAB (Natick, MA, USA). The daily geographic average was then computed from these locations and used as the central geographic location for each day.

The following section describes how the interpretations were made based on the visual inspection of the radar images. The large echo surrounding the ship's location corresponded to a blind, saturated zone, where no seabird presence was observed. Very large, slowly moving echoes with variable shapes and fuzzy contours indicate the presence of rain. Very large and stable echoes with sharp edges indicate the presence of coastlines. Boats had solid, rectangular and slow-moving (i.e. short trail) echoes. Small, erratic, widespread and weak echoes were indicators of the presence of waves. Finally, seabird echoes are typically small, of varying intensity and size, and fast-moving (relative to the boats). It is important to note that one seabird echo most likely represents a tight group of flying birds, the number of which determines the echo's size and intensity. A large single bird (for example, a skua) may create a visible echo at close distance, but not medium- to small-sized birds (i.e. noddies and terns) (Gueotec captain, personal communication).

Reviewing (>30) mosaics revealed that days could be easily classified by eye as having ‘few’ or ‘many’ birds. Therefore, each mosaic was visually scanned, and each day was classified as ‘few’ or ‘many’ for further analysis.

To test whether this subjective classification was coherent, the number of echoes was calculated for every tile as well as the median of the number of echoes for each day (excluding tiles when the radar was in standby mode). Only echoes between 3 and 50 pixels in the radar disc and further than 6.7 km (66 pixels) from the boat were considered to reduce the effects of the blind zone and large rains on the data. The ‘many’ group had 3.7 times more echoes than the ‘few’ group (median: 3 and 11 in the ‘few’ and ‘many’ groups, respectively). As an illustration of the little overlap between the two groups, the 75th percentile of the ‘few’ group corresponded to the 25th percentile of the ‘many’ group (six echoes). A more careful examination of a few days that seemed misclassified indicated that they were days containing anomalies such as high gain (lot of noisy echoes), a lot of rain (with noisy echoes on the edges of the rain fronts) or that they only had a small portion of the day showing ‘many’. The subjective classification was therefore coherent with the number of echoes in the vast majority of days. It is important to note that the number of seabird echoes calculated on single isolated tiles cannot be reliably estimated without considering the spatiotemporal coherence of echoes across consecutive frames. Visual detection allowed for a coherent and concurrent assessment of information from echoes and trails between tiles to tease apart noise from real echoes, especially on days with difficult recording conditions. Given the huge dataset used here, a simpler and more robust categorical variable obtained visually was preferred to a complex and settings-sensitive continuous variable obtained algorithmically.

The presence of various spatial patterns was noted as ‘present’ or ‘absent’ for each day. A true count would have required the observation of all the 5000+ daily images, which would be close to impossible by eye. Patterns were noted as ‘present’ only if they were observed at least once clearly without doubts. Patterns are easy to recognize and detect (figure 1), but doubt can arise from the fact that successive tiles are approximately 6 minutes apart, thus occasionally not displaying the pattern at its best visible moment. However, I am confident that I did not miss many of them because a given pattern usually occurs more than once a day, which reduces the chance of missing it on a daily scale. Thus, the daily occurrence estimates reported in this study should be treated as minima, i.e. conservative figures robust to detection.

A generalized linear mixed model (GLMM, function fitglme in MATLAB) was used to test for the effect of bird density (low versus high, fixed effect), and trip within boats (random effect) on the probability of spatial pattern occurrences (present versus absent). The effect of boats (*n* = 2, fixed effect) was initially tested, but this variable was finally removed from the final model for a constant lack of significance. The model was fitted using a binomial distribution, logit link function and Laplace approximation as a fitting method.

One spatial pattern (‘chessboard’—see Results) appeared to have a particular daily time distribution. In order to assess this, 30 days where the chessboard was observed from each of the two boats (total = 60 out of 209 days) were randomly selected, thus reducing the effects of spatiotemporal autocorrelation between consecutive days. For each of these days, the mosaic was scanned again in order to manually extract the beginning and end tiles of each chessboard pattern observed. This was done using a purpose-built code that captured the coordinates of mouse clicks and converted them directly into time of day.

## 3. Results

### (a) Data collection

A total of 485 days were visually inspected (238 and 247 from the Gueotec and Gueriden, respectively).

From these analysed days, 408 were considered ‘good’ and used in further calculations; 15.6 and 16.2% of days were flagged in the Gueotec and Gueriden, respectively. A data table is given in the supplementary material.

The tuna purse-seiners operated exclusively during the day. At night, the seabird-radars were set to standby mode (resulting in a black image with only fields visible) and the ships were usually drifting. In the early morning, fishermen set the radars to active mode and started to look for seabird echoes that could indicate the presence of tuna. They usually moved all day in search of fishing opportunities, until night fell (figure 2).

Fishing voyages lasted approximately one month (median = 27 days), allowing for large dispersal in the Gulf of Guinea.

### (b) Prevalence of seabirds

Days without seabird observations on radar images never occurred. All days could be classified as having ‘few’ or ‘many’ birds (figure 3a,b).

**Figure 3.**
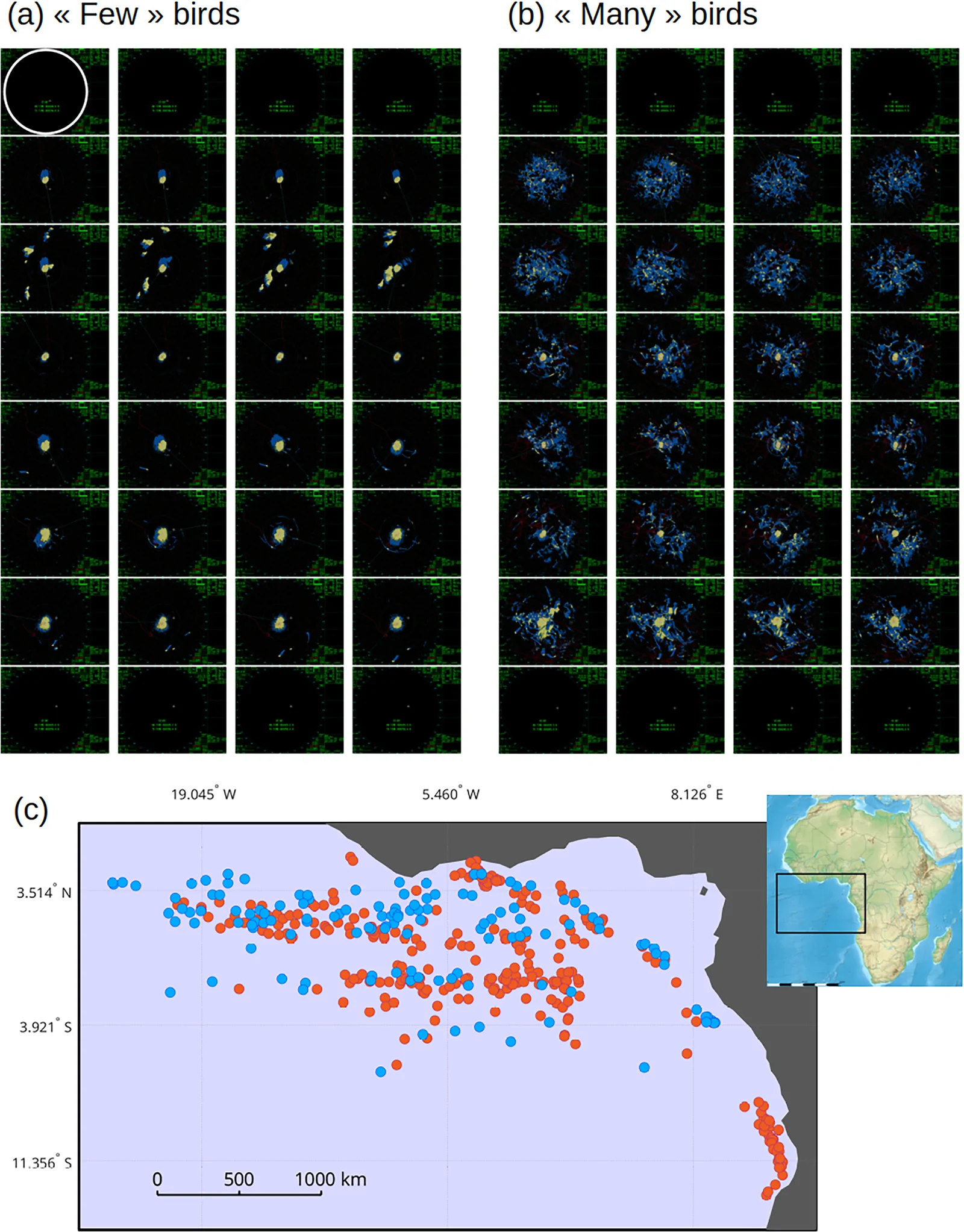
Panels (a) and (b) show zoomed portions of mosaics from two different days, with ‘few’ and ‘many’ birds, respectively. Note the presence of rain in (a) and the permanent central blind-zone around the ship (see text for details). The white circle in the first tile of panel (a) shows an approximately 30 km diameter scanned area, i.e. the radar disc. It is the same in all tiles. (c) Spatial distribution of days with ‘few’ (in blue) and ‘many’ (in red) birds estimated from the visualizations of the mosaics.

Using visual appreciation (see §2), all 408 days were classified as having few (131 days) or many (277 days) seabirds based on radar imagery.

Everywhere in the Gulf of Guinea, days with few birds were in the same areas as days with many birds, with the notable exception of days located east along the coast of Angola, offshore from the city of Luanda, which exhibited many birds (figure 3c).

### (c) Collective patterns

#### (i) Clusters

Clusters consisted of seabird echoes regrouped in space but separated by distances in the order of kilometres (figure 1a) [21]. Echoes within clusters do not show particular alignment. They can move in various directions, only keeping spatial cohesion (with inter-echoes distances being approximately 2 km) at a scale of approximately 9 km [21]. Clusters were observed on 75.3% of days (*n* = 307) and were the most commonly observed pattern. The spatial distribution of the days when clusters were observed indicates that they were observed almost everywhere (figure 4a).

#### (ii) Rakes

Rakes, i.e. the regular spacing of seabirds forming a moving line (figure 1b) [15], were observed on 68.6% of days (*n* = 280) and were also widely distributed in space (figure 4b). Echoes within rakes stand out because of their synchronicity: they align at regular intervals (approximately 500 m) and move in the same direction for up to 1–10 km [15]. Contrary to clusters, rakes form a line and move in a single direction.

#### (iii) Chessboard

Chessboard patterns, i.e. seabird echoes distributing themselves in two sets of parallel pathways oriented at approximately 90° from each other (figures 1c and 5). Contrary to rakes, echoes moving following these pathways do not synchronize to form a front of echoes moving in the same direction (i.e. as in rakes). They can move unaligned (across pathways) and in opposite directions within the same pathway. Consequently, chessboard patterns cannot be confused with clusters or two crossing rakes for example. This pattern was observed on 51.0% of days (*n* = 208) and also occurred in virtually all the Gulf of Guinea (figure 4c). The second set of lines was sometimes absent (frequency not recorded), thus showing parallel lines interspaced approximately 1–3 km (figure 5). On many occasions (frequency not measured), some seabirds moved synchronously from one line to another following right angles in the manner of a chess knight move (figures 1c (arrow) and 5). An animation of the transition between this pattern and other patterns in the morning is given in supplementary material. Interestingly, and contrary to all other patterns, they were always observed in the early mornings and/or late evenings, never in the middle of the day (figure 6).

**Figure 4.**
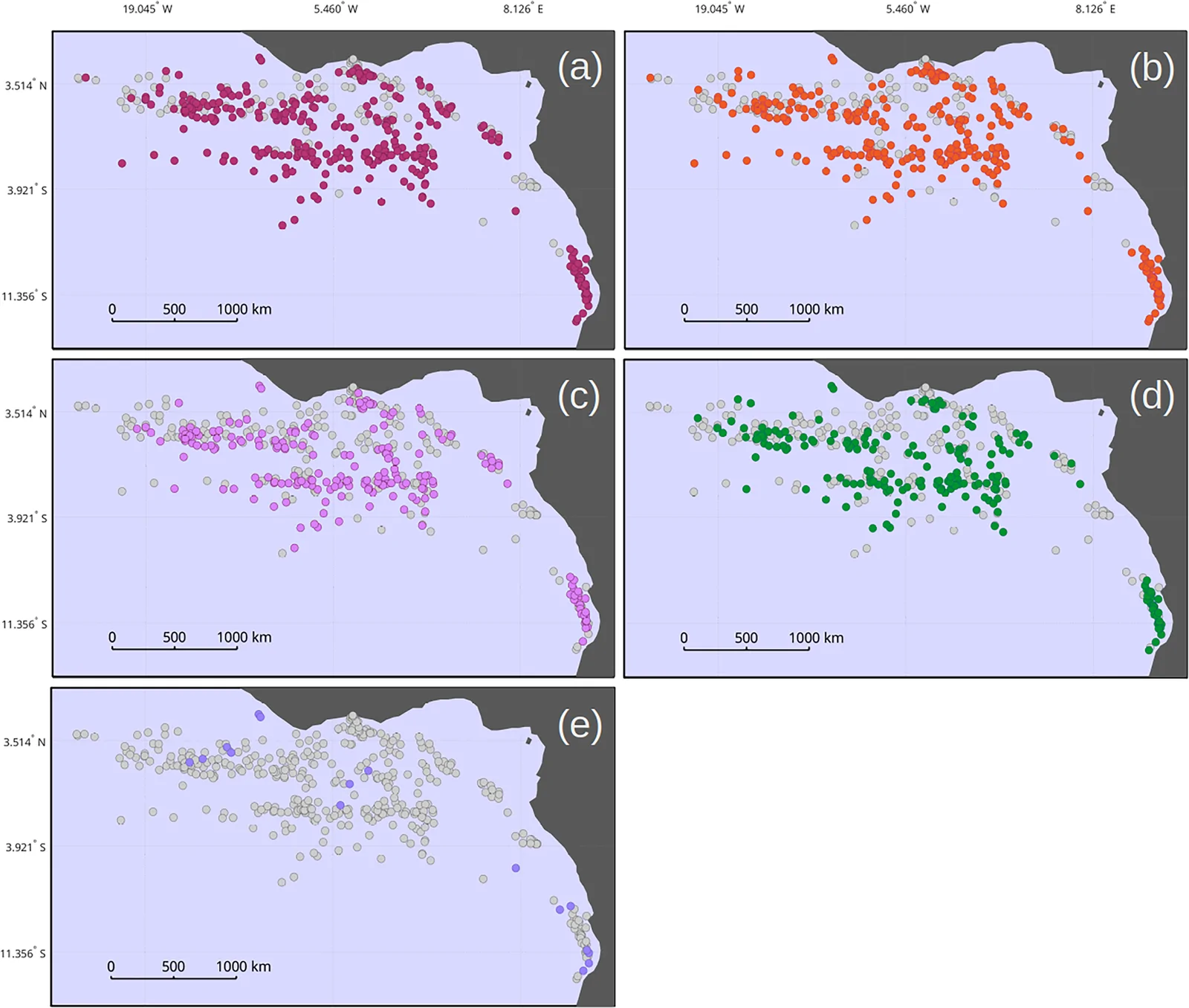
Spatial distribution of observation days when different seabirds' collective patterns were observed. (a) Clusters, (b) rakes, (c) chessboard, (d) trains and (e) parallel patterns. Note that in each map, grey and coloured dots represent days when the given pattern was not observed and was observed, respectively.

**Figure 5.**
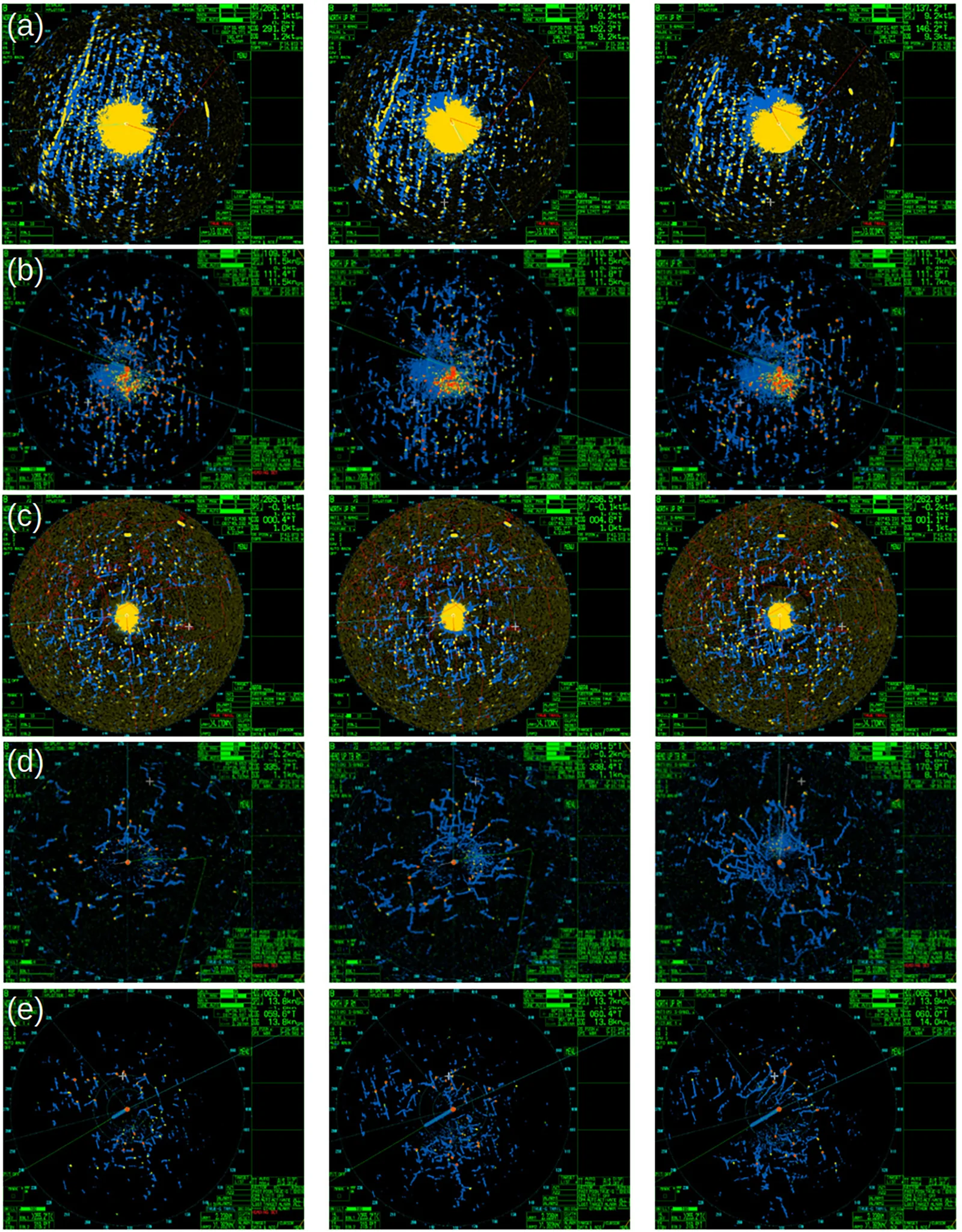
Selected radar images showing five examples (different days) of the newly discovered ‘chessboard’ pattern involving different numbers of echoes. Each row represents three images approximately 6 minutes apart of the same pattern. Note that bidirectional movements are not always present, and that when present, they are close to perpendicular. The radar disc has a 29.63 km diameter in all images.

**Figure 6.**
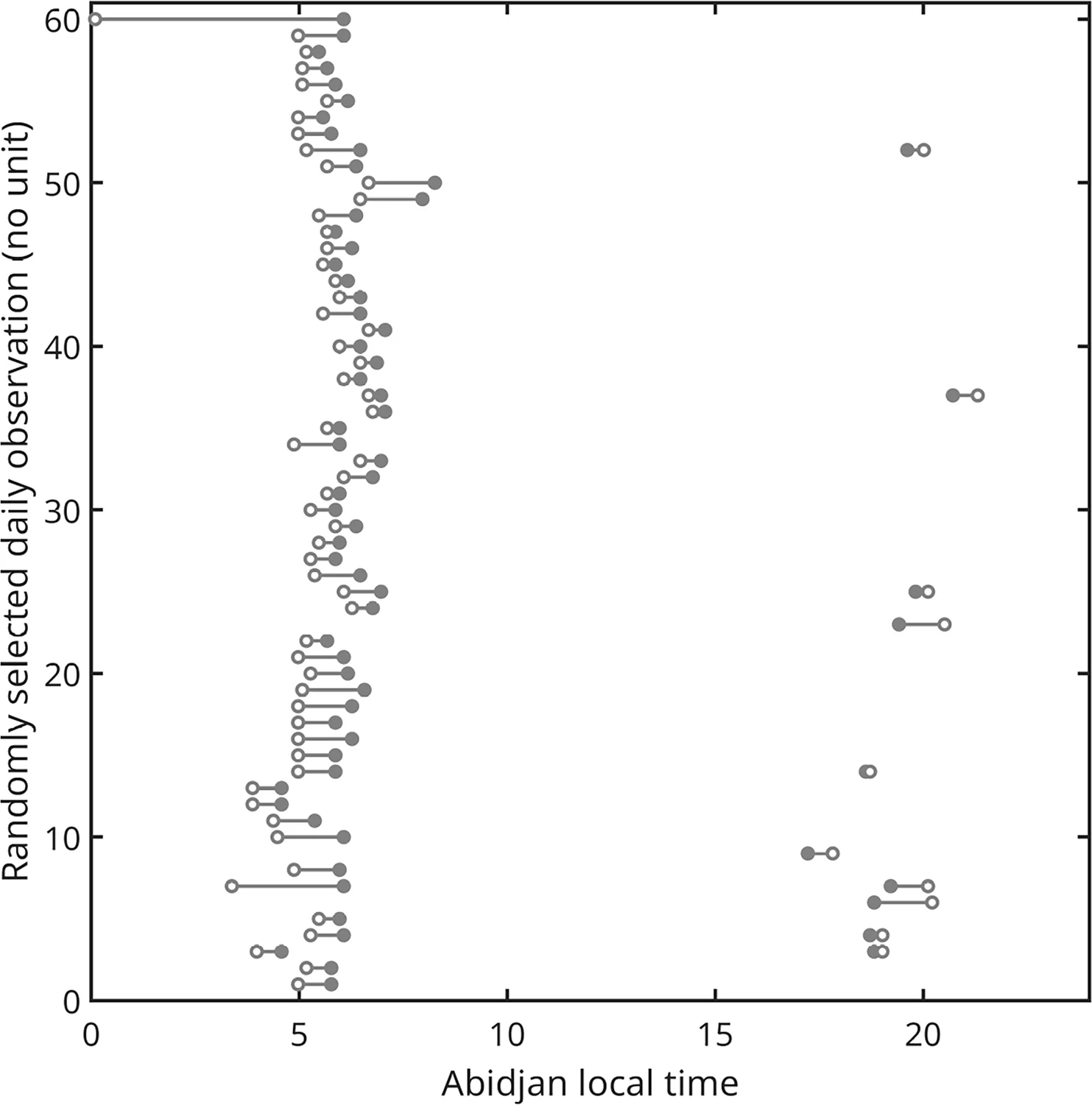
Observation time periods of the chessboard patterns in 60 randomly selected days among the days where the chessboard was observed (1–30: Gueotec ship; 31–60: Gueriden ship). Note that the white dots represent start and stop times of the radar for the day. Night times were usually not recorded (morning exception in observation 60); so, the duration of the periods is not valid. Also note that time in Abidjan could differ by ± 1 hour from the true local time as the boat moves longitudinally.

#### (iv) Trains

Trains, i.e. seabird echoes following each other over the same path over several hundred metres (figure 1d, arrow), were observed in 49.8% of days (*n* = 203) and were widely present over the Gulf of Guinea (figure 4d).

#### (v) Parallel tracks

On 18 (4.4%) days, we observed echo trajectories spaced relatively far apart (1–10 km) that shared a common bearing (figure 3e). This was the rarest pattern observed and I did not collect sufficient data to determine the large-scale spatial distribution of this behaviour (but see figure 4e).

#### (vi) Track convergences

This pattern was not initially planned for annotations as it represents a final reaction to an attractive stimulus rather than a potential collective organization pattern. However, I thought it was worth mentioning that this pattern was extremely common, often seen many times a day and virtually seen all days (not measured precisely).

### (d) Collective patterns and seabird prevalence

Bird density had a strong and highly significant positive effect on their presence probability in the cluster, rake, chessboard and train patterns, but not in the parallel pattern (table 1, OddRatios between 8 and 19.1, all *p* < 10⁻^13^). The predicted probability of presence increased from [12, 50]% under low bird density to [63, 88]% under high bird density ([min, max], table 1). Substantial among-trip variation was detected (s.d. = [0.68, 1.4], on the logit scale), confirming that trip identity explained a meaningful proportion of the residual variance independently of bird density (table 1).

**Table 1.** Main results of the GLMMs testing for the effects of bird abundance on the probability of observing the various spatial patterns observed.

spatial pattern	OddRatio	95% CI	*p*-value	prob ‘few’ (%)	prob ‘many’ (%)	among-trip variation (s.d.)
clusters	16.5	[8.5, 32.0]	1.24 × 10^−15^	50	88	1.4
rakes	19.1	[10.6, 34.5]	1.52 × 10^−20^	23	85	0.92
chessboards	8.0	[4.6, 13.7]	5.24 × 10^−13^	18	63	1.00
trains	14.3	[8.0, 25.7]	1.64 × 10^−17^	12	66	0.68
parallels	3.1	[0.6, 14.7]	0.16	1	3	1.11

Detailed results of the GLMMs for each pattern are given in the supplementary material.

## 4. Discussion

### (a) Advantages and limitations of marine radar for monitoring collective behaviour

Radar data provide an unprecedented opportunity to monitor seabird abundance and spatial organization over space and time. It is important to bear in mind that virtually none of the patterns described in this work can be visually observed from a boat, given the observation scale of the radar. Thus, a small group of seabirds engaged in a rake and flying close to a boat would likely appear to be foraging on its own, since visual observation of seabirds tends to vanish after 500 m (personal observation). Furthermore, these data allow the observation of behaviours usually studied using models, and therefore to complete and document theoretical considerations [18].

Marine radars set to observe flying seabirds provide the capability to observe collective behaviours at the community level but without species-specific information. In this study, the vast spatial and temporal coverage implies that virtually all species present in the Gulf of Guinea were recorded at some point by the radars. However, given the context of tuna associations, the most likely species involved in multi-specific aggregations in the region include sooty terns (*Onychoprion fuscatus*), Atlantic brown noddies (*Anous stolidus stolidus*), brown boobies (*Sula leucogaster*), black noddies (*Anous minutus atlanticus*), bridled terns (*Onychoprion anaethetus melanopterus*), Cory's shearwaters (*Calonectris borealis*) and Pomarine skuas (*Stercorarius pomarinus*) [24]. White-tailed Tropicbirds (*Phaethon lepturus*) and red-billed Tropicbirds (*Phaethon aethereus aethereus*) also occur in this area but tend to forage alone [25].

One possible bias in these data could arise from the fact that purse-seiners are looking for seabirds, as well as from the fact that seabirds might themselves aggregate around the boat. However, industrial tuna purse-seiners provide no discards and, given their slow speed, are unlikely to provide cues of foraging opportunities to seabirds. By contrast, fishermen spend time and resources using marine radar to locate foraging seabirds for the purposes of locating fish, which could not happen if seabirds had been heavily attracted to the ship. Accordingly, I did not observe obvious indices of seabird echoes attracted towards the ships, suggesting that if an attraction exists, it is tenuous and would require a more detailed analysis to be described. Therefore, I am confident about the fact that the presence of the fishing boat had only little (if any) effect on the data. However, the number of days with ‘many’ birds may have been over-represented, given that finding birds was the goal of the fishermen.

The presence or absence of a pattern on a given day was assessed by only one observer. Given that the patterns were quite obvious to detect, it is expected that the introduced bias in the observations was low, but they may exist and are important to consider.

### (b) Prevalence of seabirds

Despite efforts made by fishermen to look for seabirds and keep contact with them, a substantial number of days (32.1%) showed few birds. The fact that these days roughly co-occurred in space with days showing many birds illustrates that seabirds' aggregate at scales even larger than clusters and that these aggregations can change from day to day. Because many seabirds fly faster than fishing boats (cruising at approx. 20 km h^−1^), the aggregations can disorganize and reorganize in different locations more quickly than fishing boats can respond to them; therefore, purse-seiners may lose contact with large seabird aggregations, and need to constantly relocate.

The fact that days with ‘few’ and ‘many’ birds had only marginal overlap in the number of echoes suggests that seiners were either in or out of such large aggregations, and that almost no intermediate state existed at the daily scale.

This dynamic ‘large aggregation scale’, or ‘productive area’ [19], might be the only one allowing the ideal free distribution principles to hold true, i.e. the scale at which the distribution of predator abundance best matches that of their prey. A more detailed description of these large aggregations and their dynamics is needed to fully understand their characteristics.

The offshore waters of Angola were an exception, as they always showed many birds. This area might be particularly attractive to seabirds (but also to tuna) because of the presence of a rich and attractive shelf break in this area, as shown by the high diversity of seabirds recorded [26].

### (c) Collective patterns

Clusters were first described in detail in the western tropical Indian Ocean [21]. These observations, together with the wide distribution of clusters reported in the eastern tropical Atlantic, strongly suggest that clusters might involve a wide range of seabird species. Together with rakes, which have already been described in this study area [15], these two patterns stand out because of their ubiquity and appear as major structuring patterns in tropical seabird communities and are thus likely to have high adaptive values.

To my knowledge, chessboard patterns were never described before. This pattern was common and spatially widespread. Its strict occurrence at dawn and dusk suggests that it might represent a collective strategy associated with the availability of vertically migrating prey, the distribution of which is being driven by physical factors such as wind, tidal currents, internal waves, Langmuir circulation, etc. [27–29]. Alternatively, this pattern could be owing to non-foraging factors, such as social organization associated with setting up night-time rafts [30,31]. Given that this pattern can last for hours in the morning before transitioning to other types of aggregation patterns (see supplementary material) I suspect that this is most likely associated with foraging. Clearly, further investigation is needed to fully understand this pattern.

Trains have previously been described in griffon vultures (*Gyps fulvus*) [32]. In seabirds, this pattern resembles the trail-following behaviour described in colony-commuting common terns (*Sterna hirundo*) and roseate terns (*S. dougallii)* [33]. In vultures, trains were also found to be paired, thus forming rails lasting up to 10 min. However, this was not observed in the present study. Instead, trains were relatively short-lived, rarely seen over two consecutive tiles (so they lasted less than 6 minutes most of the time). I suppose that trains do not represent strategic collective organization *per se*, but rather an indirect information transfer between small groups of birds, as described in cape gannets (*Morus capensis*) [7].

Echoes forming parallel patterns appeared simultaneously but uncoordinated, which suggests that this pattern reflects migratory behaviour, unrelated to foraging. Indeed, track convergences, potentially linked with birds joining at a feeding site, never occurred while parallel patterns were ongoing and this was the only pattern in which the probability of presence was unrelated to seabird density (table 1).

### (d) Significance of collective patterns prevalence

As far as I know, this study is the first to investigate the prevalence and spatial distribution of collective flight patterns over spatial scales of more than 3000 km and for more than a year. This study builds on the growing literature documenting both single and multi-species collective foraging behaviour in seabirds [5,7,8,13,19,20,34,35] and sheds light on how common these behaviours actually are. Clusters, rakes and chessboard patterns were observed frequently throughout our 690 km^-2^ scanned area, but they did not seem to be directly related to prey capture. Instead, each of them forms a type of foraging network, allowing local enhancement to be possible. Indeed, by maintaining distances and/or flying directions compatible with keeping an eye on other foragers, seabirds collectively maintain the possibility of taking advantage of others' sensory ability to detect prey [20]. Therefore, these patterns exist before local enhancement eventually occurs. This is fundamental because they could imply more cognitively advanced abilities [36] than a simple reaction to a source stimulus, as in local enhancement. They could be an integral component of optimal foraging for ephemeral prey over spatial scales on the order of thousands of square kilometres. As a hypothesis, maintaining these patterns in a multi-specific context may require anticipation, synchronization, strategic relocation, gain-risk evaluation, understanding of other species' characteristics, etc. An alternative hypothesis is that these patterns emerge from simpler local interaction rules without any individual awareness of the global pattern. Modelling studies may help disentangle this knot in the future.

Furthermore, this study invites us to rethink the way we interpret individual-based tracking data. Since the beginning of the ‘bio-logging era’, it has been common to focus on the fine-scale behaviour of individuals, losing sight of the larger scale multi-individual processes (but see [5]). For example, the Levy-flight hypothesis proposes that Levy-flights are optimal in a heterogeneous field of prey. This was tested in two species of albatrosses using detailed tracking data [37]. Track characteristics were directly linked with the putative distribution of prey, as if the forager was alone in the field, disregarding potential collective processes.

I showed results about seabird foraging in the oligotrophic waters of the Gulf of Guinea. In such an ecosystem, prey are scarce and their availability to seabirds depends largely on the presence of sub-surface predators such as tunas and dolphins [1–4]. In this context, the collective multi-specific foraging patterns described in this study allows the maintainance of a permanent collective network of foragers able to share information, a likely evolutionary stable solution to scarce and unpredictable prey. In light of the results presented here, competition is likely secondary in the cost–benefit conundrum about seabird group foraging [19], relegating the notion of competition in feeding flocks to mere group interference. Central to this question is how shareable prey are. Multi-specific feeding flocks are regrouped on schools of prey, not individual prey; therefore, one can decently consider that ‘prey’ are highly shareable in the context of seabird group foraging.

In terms of conservation, the ubiquity of collective patterns at sea (this study) together with the ubiquity of positive (to seabirds) interactions within multi-specific associations [2] warn about the risks associated with biodiversity erosion and strongly advocate for taking into account the functional aspects brought by the whole community of marine predators, i.e. an ecosystem-based approach to protecting marine habitats. All observed patterns that are believed to be associated with foraging were significantly more likely to occur when many echoes were present. This observation potentially bridges the gap between population size and foraging efficiency of predator guilds. This suggests that an optimum may exist in population sizes that maximize foraging efficiency while minimizing competition.

Radar technology, providing the capability of scanning large areas far beyond our natural observation range, has proven to be unrivalled in moving the field towards a better understanding of the complexities of the collective behaviour of seabirds at sea.

## Supplementary Material



## Data Availability

Several mosaics at the full resolution are provided as supplementary material. Supplementary material is available online [38].

## Theme

One contribution of 14 to a theme issue ‘Multi-predator feeding aggregations in pelagic waters’.
