# Supplementary material for: Marine radar reveals novel group foraging patterns in pelagic seabirds
Source: Philos Trans R Soc Lond B Biol Sci. 2026 Oct 15;381(1961):20250203. doi: 10.1098/rstb.2025.0203 (PMC13670078; doi:10.1098/rstb.2025.0203)
Supplement: Supplementary file 1 [file rstb.2025.0203.zip › rstb.2025.0203/rstb20250203_si_003.pdf]

Marine radar reveals novel group foraging patterns in pelagic seabirds

Yann Tremblay

Suplementary materials :

Individual results of each GLMM model fitted for each patterns

ALL PATTERNS:

Model: Number of observations: 408, Fixed effects coefficients: 2, Random effects coefficients: 8, Covariance parameters: 1, Distribution: Binomial, Link: Logit, FitMethod: Laplace

Formula: response ~ 1 + bird_density + (1 | boat:trip)

CLUSTERS

Model fit statistics:

AIC	BIC	LogLikelihood	Deviance
343,96	355,99	-168,98	337,96

Fixed effects coefficients (95% CIs):

Name	Estimate	SE	tStat	DF	pValue	Lower	Upper
Intercept	-0,53966	0,54339	-0,99313	406	0,32124	-1,6079	0,52856
bird_density	2,8054	0,3367	8,3321	406	1,237e-15	2,1435	3,4673

Odds Ratios

Term	OddsRatio	CI_low	CI_high	pValue
Intercept	0,58295	0,20095	1,6911	0,32124
bird_density	16,534	8,5464	31,989	1,2374e-15

RAKES

Model fit statistics:

AIC	BIC	LogLikelihood	Deviance
380,95	392,99	-187,48	374,95

Fixed effects coefficients (95% CIs):

Name	Estimate	SE	tStat	DF	pValue	Lower	Upper
Intercept	-1.192	0.39137	-3.0458	406	0.0024721	-1.9614	-0.42266
bird_density	2.9502	0.3006	9.8144	406	1.5243e-20	2.3593	3.5412

Odds Ratios

Term	OddsRatio	CI_low	CI_high	pValue
Intercept	0.30361	0.14099	0.65381	0.0024721
bird_density	19.111	10.602	34.447	1.5243e-20

CHESSBOARDS

Model fit statistics:

AIC	BIC	LogLikelihood	Deviance
482,02	494,05	-238,01	476,02

Fixed effects coefficients (95% CIs):

Name	Estimate	SE	tStat	DF	pValue	Lower	Upper
Intercept	-1,5286	0,42516	-3,5954	406	0,0003637	2,3644	-069283
bird_density	2,0734	0,27787	7,4617	406	5,2422e-13	1,5272	2,6197

Odds Ratios

Term	OddsRatio	CI_low	CI_high	pValue
Intercept	0.21683	0.094236	0.49892	0.00036376
bird_density	7.9518	4.6125	13.709	5.2422e-13

TRAINS

Model fit statistics:

AIC	BIC	LogLikelihood	Deviance
467,27	479,3	-230,64	461,27

Fixed effects coefficients (95% CIs):

Name	Estimate	SE	tStat	DF	pValue	Lower	Upper
Intercept	-2.0002	0.358	-5.5872	406	4.2362e-08	2.704	-1.2965
bird_density	2.6609	0.2984	8.9172	406	1.6457e-17	2.0743	3.2475

Odds Ratios

Term	OddsRatio	CI_low	CI_high	pValue
Intercept	0.13531	0.067079	0.27293	4.2362e-08
bird_density	14.31	7.973	25.683	1.6457e-17

PARALLELS

Model fit statistics:

AIC	BIC	LogLikelihood	Deviance
144,57	156,61	-69,287	138,57

Fixed effects coefficients (95% CIs):

Name	Estimate	SE	tStat	DF	pValue	Lower	Upper
Intercept	-4,5397	0.85991	-5.2793	406	2.1173e-07	6.2301	-2.8493
bird_density	1.1342	0.7943	1.428	406	0.15407	0.42721	2.6957

Odds Ratios

Term	OddsRatio	CI_low	CI_high	pValue
Intercept	0.010677	0.0019791	0.057597	2.1173e-07
bird_density	3.1088	0.65535	14.748	0.15407
